# Bouveret’s Syndrome: Sense and Sensitivity

**DOI:** 10.1080/20009666.2018.1478565

**Published:** 2018-06-12

**Authors:** John Ong, Carla Swift, Sharon Ong

**Affiliations:** a Department of Engineering, University of Cambridge, Cambridge, UK; b Gastroenterology and Hepatology Training Programme, Cambridge Deanery, Cambridge, UK; c Department of Gastroenterology, Addenbrooke’s Cambridge University Hospital, Cambridge, UK; d Department of Anaesthesia and Surgical Intensive Care, Singapore General Hospital, Singapore

**Keywords:** Bouveret’s syndrome, gallstone ileus, gallstones, gastric outlet obstruction, duodenal obstruction

## Abstract

Bouveret’s syndrome is an uncommon disease characterised by gastro-duodenal obstruction caused by an impacted gallstone in the upper gastrointestinal tract. Here we submit a letter to the editor in response to an article published by Gandhi and Jani (2018) entitled ‘Rare cause of gastric outlet obstruction’ where we raise three points of contention that readers should take into consideration.

Dear Editor,

We read with interest the case report entitled ‘Rare cause of gastric outlet obstruction’ by Gandhi and Jani (2018). Though the article is concise and well written, we would like to highlight three points of contention.

Firstly, the endoscopic images provided in Figure 2 do not show a gallstone occluding the lumen of the duodenum but rather a yellowish-brown gallstone within a large ulcer with surrounding congealed blood. Similar to the endoscopic findings, the first CT scan did not demonstrate a gallstone occluding the gastric or duodenal lumen. In considering these results from both modalities, the diagnosis of Bouveret’s syndrome in this case hinges on circumstantial evidence and is open to debate. The definitive evidence that was presented; the results of the second CT scan and the laparotomy findings clearly demonstrated gallstones in the jejunum and ileum which favours the diagnosis of gallstone ileus rather than Bouveret’s syndrome. We acknowledge the images from the first endoscopy were suboptimal and it may well be that the authors have undisclosed clinical information that helped them reached their final conclusion. However, if based solely on the information that was presented, an alternative cause of the gastric outlet obstruction is also plausible, e.g., duodenal stenosis caused by inflammation around the large duodenal ulcer which is supported by the retention of congealed blood within the duodenum and the subsequent resolution of symptoms with intravenous proton pump inhibitors.

Secondly, in describing the pathogenesis of Bouveret’s syndrome the authors stated the passage of large stones through bilio-enteric fistulas result in gallstone impaction and gastric outlet obstruction. This description is however incomplete. There are three types of fistulas through which a gallstone can enter the upper gastrointestinal tract to cause Bouveret’s syndrome. More specifically, these are cholecysto-duodenal, choledocho-duodenal or cholecysto-gastric fistulas, and all three have been reported in literature on Bouveret’s syndrome. The latter was omitted by the authors. Also, despite suspecting a cholecysto-duodenal fistula in this case, the authors have not reported if one was observed on the second CT scan or during surgery.

Thirdly and most importantly, we disagree with the authors that esophago-gastro-duodenoscopy (EGD) is the gold standard of diagnosis of Bouveret’s syndrome. EGD has reported sensitivity rates of approximately 69% [1] versus CT which lies between 79.1% and 93% [2,3]. We acknowledge that 15–25% of gallstones are iso-attenuating on CT, but in such instances MRI is an alternative [4]. The diagnosis of Bouveret’s syndrome relies on a multi-modal approach to investigation, with the preferred investigation usually being radiological in the first instance and endoscopy to follow if radiological imaging is inconclusive. Radiological imaging is also fundamental to identify other, more common causes of gastric outlet and bowel obstruction which can manifest with similar signs and symptoms to Bouveret’s syndrome. Imaging is advantageous because it is non-invasive, commonly available and provides additional anatomical information which would have otherwise been missed on EGD, for example cholecysto-duodenal fistulas are visualised in only 13% of EGDs [1]. Therefore, the authors should exercise caution when describing EGD as the ‘gold standard’ for diagnosis of Bouveret’s syndrome. A diagnostic EGD performed in a patient with gastric outlet or bowel obstruction often yields poor views, increases the risk of aspiration in patients without a definitive airway, and can be potentially catastrophic in the presence of a perforated viscus.

In summary, the diagnosis of Bouveret’s syndrome is dependent on a multi-modal approach to investigation, which in turn leads to effective treatment of the disease. EGD is not considered a ‘gold standard’ investigation and it should not be a first-line investigation if Bouveret’s syndrome is suspected.

Yours sincerely,

Dr John Ong, Dr Carla Swift, and Dr Sharon Ong
